# Effector memory differentiation increases detection of replication-competent HIV-l in resting CD4+ T cells from virally suppressed individuals

**DOI:** 10.1371/journal.ppat.1008074

**Published:** 2019-10-14

**Authors:** Elizabeth R. Wonderlich, Krupa Subramanian, Bryan Cox, Ann Wiegand, Carol Lackman-Smith, Michael J. Bale, Mars Stone, Rebecca Hoh, Mary F. Kearney, Frank Maldarelli, Steven G. Deeks, Michael P. Busch, Roger G. Ptak, Deanna A. Kulpa

**Affiliations:** 1 Southern Research, Frederick, Maryland, United States of America; 2 Department of Pediatrics, Emory University, Atlanta, Georgia, United States of America; 3 HIV DRP, NCI at Frederick, NIH, Frederick, Maryland, United States of America; 4 Vitalant Research Institute, San Francisco, California, United States of America; 5 Department of Laboratory Medicine, University of California, San Francisco, San Francisco, California, United States of America; 6 University of California, San Francisco (UCSF), San Francisco, California, United States of America; UNC-Chapel Hill, UNITED STATES

## Abstract

Studies have demonstrated that intensive ART alone is not capable of eradicating HIV-1, as the virus rebounds within a few weeks upon treatment interruption. Viral rebound may be induced from several cellular subsets; however, the majority of proviral DNA has been found in antigen experienced resting CD4+ T cells. To achieve a cure for HIV-1, eradication strategies depend upon both understanding mechanisms that drive HIV-1 persistence as well as sensitive assays to measure the frequency of infected cells after therapeutic interventions. Assays such as the quantitative viral outgrowth assay (QVOA) measure HIV-1 persistence during ART by *ex vivo* activation of resting CD4+ T cells to induce latency reversal; however, recent studies have shown that only a fraction of replication-competent viruses are inducible by primary mitogen stimulation. Previous studies have shown a correlation between the acquisition of effector memory phenotype and HIV-1 latency reversal in quiescent CD4+ T cell subsets that harbor the reservoir. Here, we apply our mechanistic understanding that differentiation into effector memory CD4+ T cells more effectively promotes HIV-1 latency reversal to significantly improve proviral measurements in the QVOA, termed differentiation QVOA (dQVOA), which reveals a significantly higher frequency of the inducible HIV-1 replication-competent reservoir in resting CD4+ T cells.

## Author summary

Quantifying the number of cells harboring HIV-1 provirus is critical to evaluating HIV cure interventions, but precise quantification of the latent reservoir has proven to be technically challenging. Our data demonstrates that targeted differentiation of CD4+ T cells to an effector memory phenotype is a successful strategy for promoting latency reversal *in vitro*, and significantly enhances the performance of existing protocols to quantify the frequency of replication-competent HIV-1 in resting CD4+ T cells from virally-suppressed individuals. Using peripheral blood samples from well-established and characterized cohorts, we show the presence of a significantly higher frequency of replication-competent provirus than has previously been reported, raising the average estimated frequency 18-fold over the established QVOA measures. These results demonstrate that *ex vivo* effector memory differentiation has moved reservoir measurements closer to what may be the bona fide inducible replication-competent reservoir frequency, thus beginning to bridge the gap between viral outgrowth and molecular-based quantification. Taken together, these data support accumulating evidence that effector memory differentiation is a key pathway to HIV-1 latency reversal that may be exploited for assay development, mechanistic understanding, and therapeutic interventions.

## Introduction

ART suppresses HIV-1 replication to undetectable levels but cannot eliminate the virus due to early establishment of a persistent reservoir of latently infected cells that provides a long-lived source of rebound viremia [1–4]. The mechanisms that govern latency reversal and viral rebound *in vivo* are still being defined, including the elucidation of the cellular compartments that contribute to HIV-1 reactivation after ART interruption [5–12]. Understanding the mechanisms that maintain or reverse latency *in vivo* is critical for the success of therapeutic strategies aimed at supporting viral remission, controlled treatment interruption, or cure.

Viral rebound may originate from several cellular subsets, including naive CD4+ T cells and myeloid cells; however, the majority of proviral HIV-1 DNA persists in CD4+ T cells displaying a memory phenotype, which include central (T_CM_), transitional (T_TM_) and effector (T_EM_) memory subsets that are each endowed with distinct phenotypic and functional properties and can persist for decades [13–19]. The latent reservoir frequency has been estimated to be approximately one in one million resting CD4+ T cells but can be highly variable among successfully treated individuals [20]; influenced by the nadir CD4^+^ T cell count [21], the CD4/CD8 ratio [22], the time between infection and initiation of ART [13] and the total time on ART [23]. Quantification of the frequency of cells with intact provirus is a critical component in understanding HIV pathogenesis under ART, as well as the ability to evaluate therapeutic cure strategies to eliminate the latent reservoir. A number of approaches have been developed to quantify the HIV-1 reservoir from *ex vivo* peripheral blood (reviewed in [24]), including molecular based assays to quantify cell-associated HIV-1 RNA [25–27] or HIV-1 DNA frequencies [28–33] or both [34], along with assays that specifically assess the replication-competent reservoir through quantitative viral outgrowth [5, 23, 32, 35–39]. Importantly, reservoir quantification approaches to date have shown advantages as well as limitations relating to either over or under estimation of the replication-competent reservoir size, the inability to distinguish intact versus defective proviruses, or low throughput for clinical applications.

Another significant challenge inherent in these assays is the translation of *ex vivo* measurements to the replication-competent reservoir that may be inducible *in vivo*, both in the context of viral rebound after treatment interruption as well as during targeted latency reversal as a therapeutic intervention. Multiple studies have identified pathways that support HIV latency reversal in CD4+ T cells, including activation of protein kinase C, NFAT, and NF-ΚB signaling as well as changes in epigenetic modifiers [40–42]. Expression of these pathways is associated with cellular activation, and have been shown to support HIV-1 latency reversal *in vitro* [40, 43–46]. However, several studies have demonstrated that cellular activation alone may not be sufficient to induce latency reversal from a signficant proportion of replication-competent proviruses from virally suppressed individuals [47, 48]. Indeed, recent data suggest a role for the acquistion of effector function as a pathway to more effective HIV-1 latency reversal in memory CD4+ T cells [49]. Differentiation of memory CD4+ T cells results in the acquistion of effector function, including effector cytokine production (IFN-γ, TNF-α, IL-2), and also includes the upregulation of activation markers (CD38, CD69, IL2Rα/CD25), transcription factors known to activate HIV-1 (NFAT, STAT5, NF-ΚB, E2), and the translation apparatus (EIF2S1, EIF4A1; [50–53]; reviewed in [54–56]). Effector memory differentiation also leads to changes in the machinery controlling epigenetic modulation, such as histone deacetylases (HDAC1, HDAC3, HDAC4, HDAC8, HDAC9) and the chromatin remodeling complex (SMARCAL1, SMARCD1, SMARCE1) [57–63]. As expected, these changes in gene expression correlate with and predict HIV-1 latency reversal in quiescent CD4+ T cell subsets like T_CM_ [49]. However, these studies were performed employing assays like TILDA, which quantifies the frequency of the inducible reservoir by measuring multi-spliced HIV RNA expression in mitogen activated total CD4+ T cells as a surrogate for virus production [64]. The observed correlation between the acquisition of an effector memory phenotype and HIV-1 latency reversal led us to hypothesize that effector memory polarization is an efficient pathway to latency reversal of replication-competent HIV-1 in lymphoid reservoirs. To test this hypothesis, we examined the impact of effector memory differentiation in *ex vivo* resting CD4+ T cells in the context of the quantitative viral outgrowth assay (QVOA).

## Results

### Effector memory differentiation enhances latency reversal of the replication-competent reservoir

**Fig 1:**
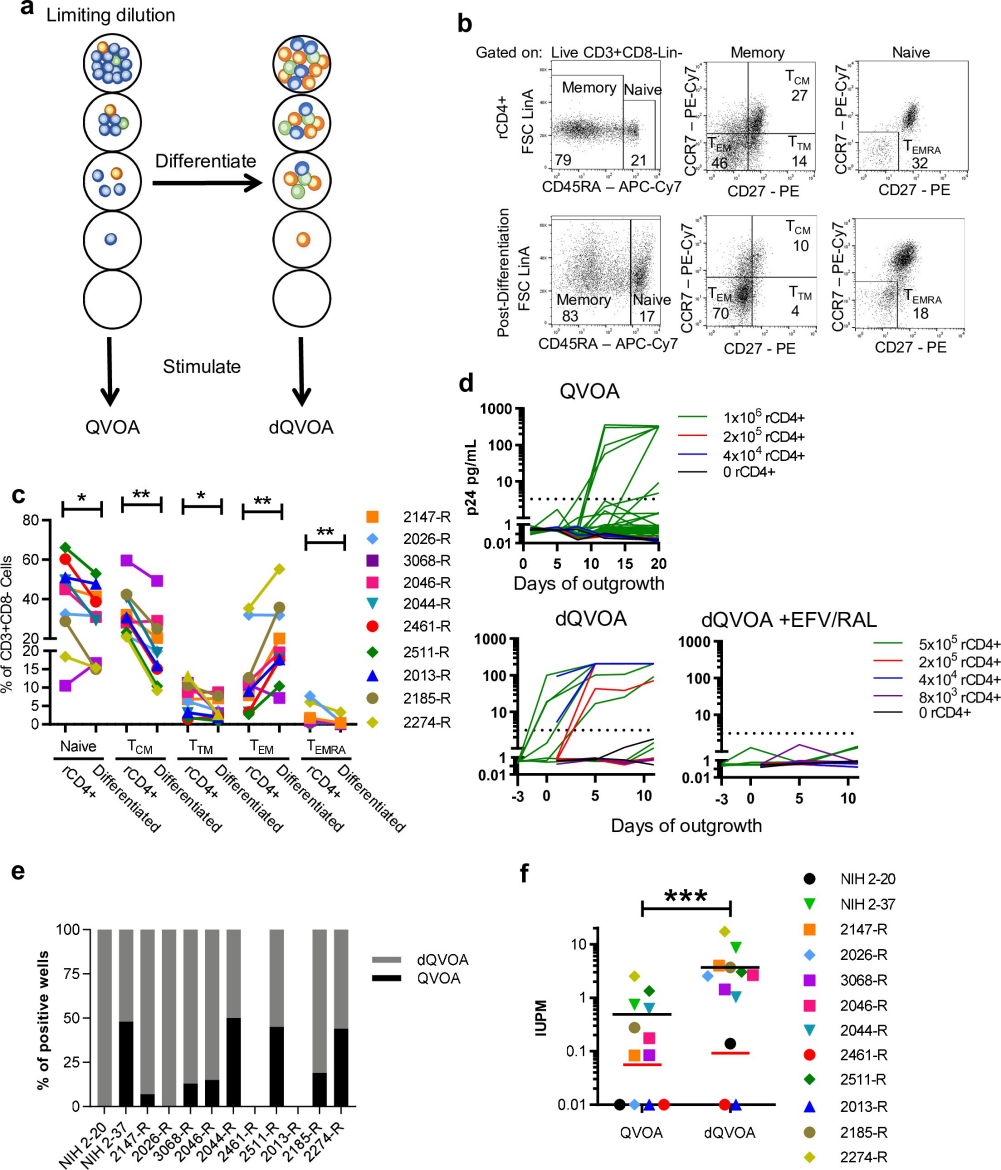
*Ex vivo* differentiation of resting enriched CD4+ T cells prior to QVOA increases detectable reactivation of latent HIV-1. **a**, Schematic of assay outline for QVOA and differentiation QVOA (dQVOA). The relative distribution of central memory (T_CM_), effector memory (T_EM_), and transitional memory (T_TM_) cells during each phase of the assays are represented as blue, orange, and green characters. **b**, Flow cytometric analysis showing the memory T cell subset gating strategy on samples from RAVEN participant 2274-R either after resting enrichment (top panel, cells that enter QVOA) or after 7 days of differentiation (bottom panel, cells that enter dQVOA). The numbers in each gate represent the percentage of events arising from its parental population. **c**, Column plot showing the proportions of naïve, T_CM_, T_TM_, T_EM_ and T_EMRA_ cells as a percentage of live CD3+Lineage-CD8- lymphocytes before and after 7 days of differentiation. NIH 2–20 and NIH 2–37 were assessed for memory subset distribution using a different flow cytometry antibody panel compared to the RAVEN participant samples and therefore were not included in this analysis. **d**, XY plots showing HIV-1-Gag expression level in each well from QVOA (top panel) and dQVOA (bottom panels) over time. The rCD4+ T cell source was RAVEN participant 2147-R. Stimulation was performed on day 0 for QVOA and dQVOA and when noted ART (EFV/RAL) was included in all media in the assay. Limiting dilutions performed for each assay type are shown. **e**, Bar graph showing the proportions of QVOA wells (black) found to be HIV-1-Gag+ compared to dQVOA wells (grey). **f**, Column graph showing the latent HIV-1 reservoir measured as infectious units per million rCD4+ T cells (IUPM) through QVOA and dQVOA. Limit of detection for each assay is represented by red bars. Wilcoxon matched-pairs signed rank tests were used and * denotes p < 0.05, ** denotes p < 0.01, and *** denotes p < 0.001.

To characterize the impact of effector memory differentiation on induction and outgrowth of replication-competent HIV-1 in resting CD4+ T cells in the context of the QVOA [36], we employed a combination of γ-chain (IL-7, IL-15) and dendritic cell (DC)-derived (IL-6, IL-10, TNF-α) cytokines previously established to polarize memory CD4+ T cells to acquire effector function and downregulate chemokine receptor CCR7 (Fig 1A; [65]). Initially, resting CD4+ T cells (rCD4+) were enriched from cryopreserved PBMCs from 12 virally suppressed research study participants (Table 1), 10 from the RAVEN cohort (median time of suppression 14.5 years, range 10.4–19.3) and 2 from an NIH cohort (median time of suppression 16.5 years, range 16.1–16.8), and the proportion of cells in the naïve compartment and each memory CD4+ T cell subset (T_CM_, T_TM_, and T_EM_) including the terminally differentiated T_EM_ subset that has re-gained CD45RA expression (T_EMRA_) was determined (Fig 1B and 1C).

**Table 1:** HIV-Infected participant characteristics.

Participant ID	Age	Sex	CD4+ T cells (Cells/mL)	Plasma VL (Copies/mL)	ART regimen at time of collection	Minimum length of viral suppression (Years)
NIH 2–20	65	M	638	<40	ABC/3TC/DTG	16.1
NIH 2–37	62	M	792	<40	TDF/FTC/RAL	16.8
2147-R	59	M	497	<40	RPV/TDF/FTC	11.6
2026-R	61	M	366	<40	ABC/DTG/3TC	15.1
3068-R	62	M	472	<40	ABC/3TC, ETV, RAL	14.1
2046-R	51	M	747	<40	ECV, EFV/TDF/FTC	17.0
2044-R	65	M	486	<40	ABC/3TC, DTG	16.4
2461-R	62	M	667	<40	DTG, RPV	16.2
2511-R	48	M	397	<40	EFV/TDF/FTC, RAL	10.4
2013-R	68	M	634	<40	ABC/3TC, RAL	19.3
2185-R	59	M	624	<40	EFV/TDF/FTC	12.3
2274-R	55	M	344	<40	NVP, FTC/TDF	13.1

Clinical characteristics of participants from the NIH or RAVEN (-R) cohort are shown. Abbreviations: 3TC–lamivudine, ABC–abacavir, DTG–dolutegravir, ECV—entecavir, EFV–efavirenz, ETV–etravirine, FTC–emtricitabine, NVP–nevirapine, RAL–raltegravir, TDF–tenofovir disproxil fumarate.

Due to the fact that differentiation cytokines may induce proliferation that result in expansion or redistribution of the HIV-infected cell population, rCD4+ were distributed in limiting dilution at assay initiation, prior to all differentiation and stimulation steps (Fig 1A). Once plated in limiting dilution, the cells were never redistributed at any other point in the assay, thus preserving the original frequency of intact provirus for maximum likelihood calculations. Cells in limiting dilution (5x10^5^, 2x10^5^, 4x10^4^ and 8x10^3^ cells/well; S1 Table) were cultured in the differentiation cytokines for 7 days (Fig 1A). After differentiation culture, one well of the rCD4+ T cells was assessed to monitor for changes in CD45RA, CCR7, CD27 and IL-2Rα (CD25) expression by flow cytometry (Fig 1B). Naïve CD4+ T cells (mean fold-change based on relative percentages = 0.83±0.19; p value = 0.0195) along with the T_CM_ (mean fold-change = 0.69±0.20; p value = 0.0039), T_TM_ (mean fold-change = 0.85±0.38; p value = 0.0195) and T_EMRA_ (mean fold-change = 0.26±0.22; p value = 0.002) subsets all showed a significant decrease in frequency after differentiation culture, while the T_EM_ (mean fold-change = 2.52±1.29; p value = 0.0098) showed a significant increase (Fig 1B and 1C). The proportion of cells expressing IL-2Rα (CD25), induced by exposure to DC-derived cytokines, was significantly increased in naïve, T_CM_, T_TM_, and T_EM_ (S1A Fig). After differentiation culture, rCD4+ T cells were maintained in their original limiting dilution distribution and IL-2 and the mitogen phytohemagglutinin (PHA) were added. IL-2 plus PHA activation was performed in the presence of γ-irradiated allogeneic PBMCs at a ratio of 1:10 as previously described [36] and cultures were maintained for an additional 11 days. For viral outgrowth, the participant’s own differentiated and activated CD4+ T cells readily expanded the replication-competent reservoir, eliminating the need for addition of allogenic donor lymphoblasts as target cells that may confound replication dynamics and consequent IUPM frequency. Indeed, Geginat et al. showed previously that differentiation of T_CM_ into T_EM_ results in polarization toward Th1 and Th2 phenotypes and increased surface expression of CCR5, an HIV co-receptor that may enhance the ability of the differentiated CD4 T cells to propagate virus [65]; similar post-differentiation IFN-γ and IL-4 production and upregulation of CCR5 were confirmed using samples from HIV-infected participants 2147-R, 2511-R, and 2185-R (S1B Fig). As a control for effector memory polarization on priming resting CD4+ T cells for induction of replication-competent HIV-1 expression, rCD4+ T cells from the same participants were cultured in parallel after distribution in limiting dilution (1x10^6^, 2x10^5^, 4x10^4^ cells/well; S1 Table) with PHA plus IL-2 activation using the classical QVOA procedure previously described (Fig 1A; [36]). Per published protocol, control QVOA cultures also included the addition of γ-irradiated allogeneic PBMCs as well as activated allogeneic lymphoblasts as permissive targets for HIV-1 expansion [36]. Culture supernatants from individual wells from both the QVOA and differentiation culture QVOA (dQVOA) were collected and assessed for outgrowth of replication-competent HIV-1 by scoring for the exponential increase in supernatant HIV-1-Gag expression by p24 ELISA (Fig 1D). By inducing effector memory differentiation, we found the frequency of HIV-1-Gag+ wells detected was greatly increased from each participant across all dilutions (Fig 1D and 1E; S1 Table), resulting in an average 18-fold increase in the calculated frequency of Infectious Units Per Million rCD4+ T cells (IUPM; [66]) evaluated by dQVOA (5.203 mean IUPM) over control QVOA (0.729 mean IUPM; Fig 1F; S1 Table). The magnitude and kinetics of HIV-1-Gag detection in positive culture wells demonstrated evidence of replication-competent virus outgrowth in both QVOA and dQVOA (Fig 1D). Replication competence was further verified by including the non-nucleoside reverse transcriptase inhibitor efavirenz (EFV) and integrase strand-transfer inhibitor raltegravir (RAL) in dQVOA and monitoring the culture supernatant in each well over time (Fig 1D). We found no detectable HIV-1-Gag expression in the presence of the anti-retroviral drugs, indicating that viral release without outgrowth was not a significant contributor to the increased frequency of HIV-1-Gag+ detection in the dQVOA.

### Effector memory differentiation lowers variability in measured reservoir frequencies

Viral outgrowth assays typically employ target cells to support virus expansion using activated allogeneic lymphoblasts or permissive cell lines [37, 67–70]. However, the HIV-infected individual’s own CD4+ T cells may generally represent a target cell population that can more effectively replicate virus than allogeneic HIV-naïve donor lymphoblasts. To examine this question, we performed parallel QVOA using the standard QVOA conditions, dQVOA with target lymphoblasts added, and dQVOA (no target lymphoblasts added) on resting CD4+ T cells from three independent participants in this study (S2 Fig). In a comparison between dQVOA with and without added target lymphoblasts, two of the three participant samples showed a small increase in IUPM when no target lymphoblasts were added to the cultures, but these differences were not statistically significant. In one donor (NIH 2–20), the addition of target lymphoblasts showed a small increase in IUPM when allogeneic target lymphoblasts were added, but again overlapping confidence intervals suggest this difference is not statistically significant. By contrast, dQVOA IUPM values obtained either with or without addition of activated target lymphoblasts resulted in a higher reservoir frequency than standard QVOA, including one donor from which no measurable replication-competent virus could be detected even with repeated attempts using standard QVOA conditions (NIH 2–20; S2 Fig). Together these data suggest that while the participant’s own activated CD4+ T cells may provide a slight growth advantage to some proviruses, overall the dQVOA supports more favorable conditions to induce virus expression.

**Fig 2:**
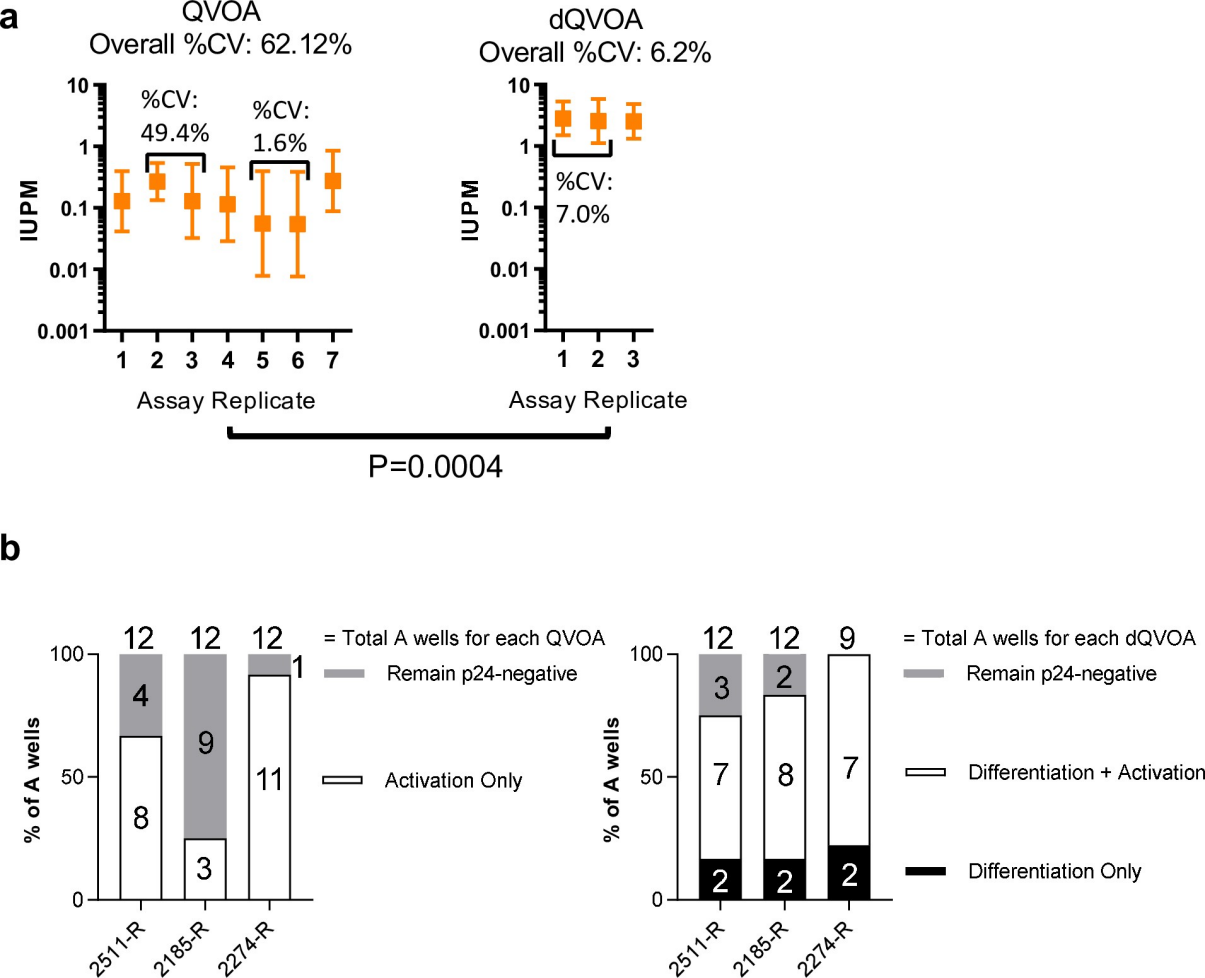
*Ex vivo* differentiation drives HIV-1 reactivation in a reproducible manner. **a**, Column plot showing the measured IUPM for QVOA (n = 7) and dQVOA (n = 3) performed on RAVEN participant 2147-R. Error bars are 95% confidence intervals generated by the program IUPMStats (http://silicianolab.johnshopkins.edu). %CV represents the coefficient of variation for each assay type. (p = 0.0004; unpaired t test with Welch’s correction). Black brackets indicate assays initiated on the same day and performed in parallel. %CV over each bracket represents the %CV for each batched assay set-up. **b**, Column plots showing the frequency of p24 positive wells after complete QVOA (left panel, white bars), differentiation alone as part of dQVOA (right panel, black bars), or after differentiation, activation, and outgrowth in complete dQVOA (right panel, white bars). For both panels, gray bars indicate the frequency of wells that remain p24 negative after completion of each assay. Frequency of HIV-1-Gag+ wells was determined using the top assay dilution for each assay.

Studies of the HIV-1 reservoir have shown that a significant proportion of integrated proviruses detected using PCR-based approaches are genetically defective, resulting from accumulations of reverse transcription errors and G-to-A mutations induced by APOBEC3G [47, 71]. Consequently, the frequency of replication-competent HIV-1 provirus is significantly lower than the frequency measured in PCR-based assays. However, the QVOA has been demonstrated to under-represent the HIV-1 reservoir due to stochastic induction of latency reversal in the context of mitogen-directed activation of rCD4+ T cells [47]. Transcriptionally-quiescent subsets like T_CM_ may harbor intact proviruses at genomic locations influenced by epigenetic modifications that are repressive to HIV-1 expression. Sequestration of transcription factors may also ultimately limit the propagation of infectious HIV-1 using standard *in vitro* culture stimuli [72]. CD4+ T cell phenotype during initiation of culture conditions in assays like QVOA may heavily influence the qualitative and temporal responsiveness to stimulation, leading to an apparent stochastic latency reversal frequency that leaves many intact proviral genomes uninduced [47]. Indeed, replicate QVOA (n = 7) using the same peripheral blood sample from RAVEN participant 2147-R in the context of PHA plus IL-2 activation alone showed a half-log range in IUPM frequencies (0.054–0.275 IUPM) with a high coefficient of variation (62.12%; Fig 2A), suggesting an inherent variability in the ability to reproducibly induce HIV-1 expression and viral outgrowth [69]. By contrast, inducing effector memory differentiation prior to activation in dQVOA not only revealed a significantly higher replication-competent reservoir frequency in this participant (2.524–2.821 IUPM; Fig 2A), an 18-fold increase in the latent reservoir size, but this increase was coupled with a 10-fold lower coefficient of variation (6.21%). An advantage of an increased frequency of positive wells observed in dQVOA (Fig 1E) is the reduction in the coefficient of variation that is generated through the maximum likelihood calculation. Together, these data demonstrate that driving effector memory differentiation supports more reproducible HIV-1 reactivation, thus reducing reliance upon stochastic mechanisms to induce HIV-1 expression.

Previous studies have suggested that γ-chain cytokines like IL-7 alone induce homeostatic proliferation, but have reported conflicting abilities to reactivate latently-infected memory CD4+ T cells [73–75], whereas IL-15 is a known inducer of latency reversal [76]. The collection of supernatant samples from dQVOA prior to PHA plus IL-2 activation allowed us to assess whether differentiation with a combination of γ-chain and DC-derived cytokines alone was sufficient to induce efficient latency reversal in rCD4+ T cells (Fig 1D; Fig 2B). We determined the frequency of positive wells at the top assay dilution from three participants in the RAVEN cohort after 7 days of differentiation culture and again after PHA activation and an additional 11 days of outgrowth, and compared these values to the frequency of positive wells at the top assay dilution generated in QVOA with PHA plus IL-2 activation alone (up to 20 days of outgrowth after PHA activation). Interestingly, we observed detectable p24 positive wells in all participants after differentiation culture alone in dQVOA (Fig 2B). Culturing differentiated rCD4+ T cells after activation further increased the number of wells exhibiting exponential viral outgrowth over differentiation alone in all 3 participants, resulting in a consistently higher measured reservoir frequency than observed from the QVOA. These results suggest that exposure to γ-chain and DC-derived cytokines alone *in vitro* can induce HIV-1 expression from latently-infected cells but is insufficient for latency reversal of a proportion of the latent reservoir unless coupled with an activating signal.

### dQVOA IUPM correlate with key clinical parameters of HIV pathogenesis

**Fig 3:**
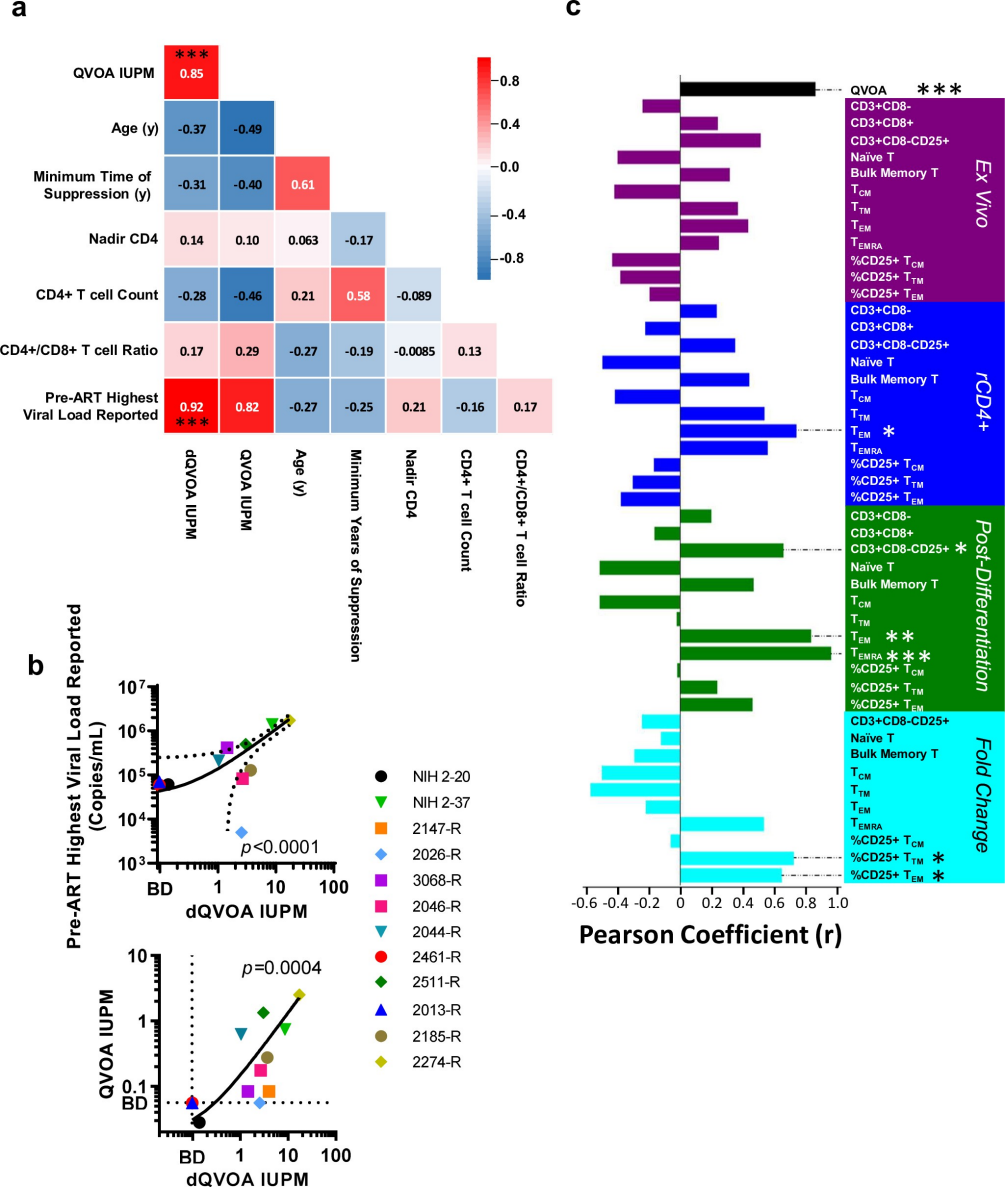
Reservoir measurements derived from dQVOA directly correlate with QVOA, pre-ART highest viral load reported, and effector memory T cells. **a,** The cross-correlation matrix (Pearson r value) of clinical measurements from linear regression. Analysis includes all available data from participants across the NIH and RAVEN cohort. *** denotes p < 0.001. **b,** XY Graphical representation of correlations found to be significant in panel **a**. **c**, Correlation with dQVOA IUPM from linear regression (Pearson r value) for each individual measurement. Analysis includes available data from participants in the RAVEN cohort. * denotes p < 0.05; ** denotes p < 0.01; *** denotes p < 0.001.

We next determined if the frequency of the HIV-1 reservoir in dQVOA correlated with measured clinical parameters in the RAVEN and NIH cohorts (Fig 3A). We performed cross-correlation linear regression analyses between participant age, duration of viral suppression under therapy, nadir CD4+ T cell count prior to ART initiation, CD4+ T cell count and CD4+:CD8+ T ratio at time of leukapheresis, and the highest viral load (VL) reported prior to initiation of ART. The dQVOA IUPM frequency in these participants correlated with that from QVOA (Spearman r = 0.85, p = 0.0004), supporting a relationship between the measured reservoir that is reactivated through activation alone and the reservoir size that is detected through effector memory differentiation pathways (Fig 3A and 3B). The IUPM frequency in rCD4+ T cells after dQVOA correlated with the highest reported pre-ART VL (Spearman r = 0.92, p = 6.3x10^-5^), suggesting higher VL prior to treatment results in greater seeding and/or retention of replication-competent provirus after initiation of ART (Fig 3A and 3B).

The magnitude of the latent replication-competent reservoir measured in dQVOA was compared to *ex vivo* lymphocyte, rCD4+ T cell, and post-differentiation parameters by linear regression (Fig 3C). The frequency of cells in the T_EM_ subset in the rCD4+ T cell population correlated with dQVOA IUPM (p = 0.013), though the T_EM_ subset in the *ex vivo* population did not correlate with statistical significance. These results are consistent with observations that the T_EM_ subset is a significant source of infected cells with intact proviruses [19, 77]. The dQVOA reservoir size also correlated with several post-differentiation populations: the CD3+CD25+ compartment (p = 0.0389), and the T_EM_ and T_EMRA_ subsets (p = 0.002 and p = 7.58x10^-6^ respectively). These data support that both activation and effector memory differentiation are key correlates of latency reversal in rCD4+ T cells. Indeed, the fold-change in frequency of the CD25+T_TM_ and CD25+T_EM_ compartment also exhibited significant correlation with dQVOA IUPM (p = 0.0182 and p = 0.0427 respectively). Identifying these correlates of latency reversal provides the basis for mechanistic understanding of factors that govern the replication-competent HIV-1 reservoir both *in vitro* and *in vivo*.

### dQVOA reveals population of expanded proviral clones in rCD4+ T cells

**Fig 4:**
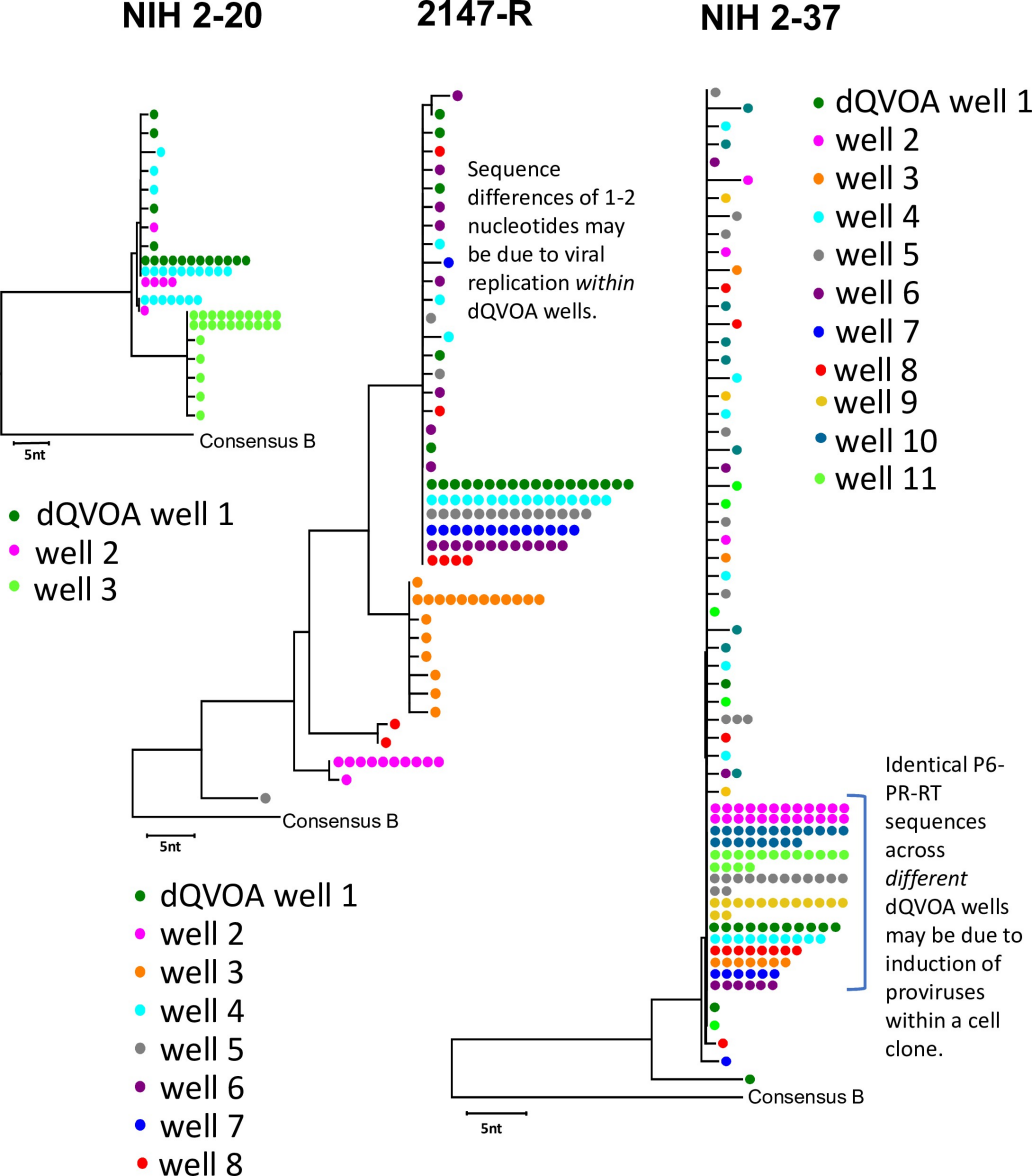
Differentiation drives reactivation of clonally-expanded, replication-competent HIV-1. P6-PR-RT single-genome sequencing[78] was performed on HIV-1-Gag+ culture supernatants from dQVOA on NIH participant 2–20 (n = 2), NIH participant 2–37 (n = 1), and RAVEN participant 2147-R (n = 1). Circles represent single RNA genomes in the supernatants. Sequences obtained from different wells are shown in different colors. Identical sequences across different wells may result from latency reversal of infected cell clones. Sequences within wells that are different by a single nucleotide or two from a large rake of identical sequences are likely the result of RT error upon viral replication in the QVOA wells rather than from induction of different proviral variants.

To characterize the viral variants induced by differentiation in dQVOA, we performed single-genome sequencing on viral RNA found in the HIV-1-Gag+ supernatants from 3 participants (Fig 4)[78]. We obtained 67 virus sequences from NIH participant 2–20, 184 from NIH participant 2–37, and 131 from RAVEN participant 2147-R. These data demonstrate that multiple variants, including probable expanded clones, were expressed from the rCD4+ populations, further supporting that differentiation conditions are inducing the expression and outgrowth of a repertoire of replication-competent HIV-1. Single-genome sequencing was performed using the P6-PR-RT primer set, which has been demonstrated previously to have a high clonal prediction score for accuracy in identifying clonal expansion in viral outgrowth assays when used on samples from donors with high HIV diversity [9, 79]. Strikingly, in the sampling performed as part of SGS analysis, each participant showed evidence of identical sub-genomic sequences across *different* dQVOA wells, suggesting that these sequences may have resulted from induction of replication-competent provirus in expanded T cell clones *in vivo*. These data are in agreement with several recent studies suggesting one mechanism contributing to HIV-1 persistence *in vivo* is homeostatic proliferation of infected memory CD4+ T cells resulting in clonal expansion [80–84]. Of note, identical sequences *within* dQVOA wells likely result from a single infected cell that was induced to expand and produce virus particles *ex vivo* and are *not* indicative of clonal expansion *in vivo*. In addition to the observation that identical sequences were found across different dQVOA wells, we also observed sequences that were different by 1–2 nucleotides from the consensus within each dQVOA well. These minor variations are a strong indicator of viral replication in the assay as expected.

## Discussion

The mechanisms responsible for the persistence of the latent HIV-1 reservoir in memory CD4+ T cell subsets *in vivo* are still being investigated, which has hindered design of effective eradication strategies. Data from SIV-infected macaques demonstrated that the viral reservoir is present in T_CM_ and T_TM_ residing in lymph nodes and gastrointestinal mucosa by day 3 post infection, suggesting seeding of the reservoir in these subsets is a very early event in infection that persists after introduction of ART [3]. Treatment interruption studies also have demonstrated viral rebound emerges from a limited pool of latent viruses, with the potential sources of rebound viremia observed after different therapeutic interventions still under investigation [6, 83, 85, 86]. The proposed dynamics of HIV-1 reservoir reactivation *in vivo* include the TCR recognition of cognate antigen by latently HIV-1-infected memory CD4+ T cells through presentation by APCs, which triggers proliferation and effector memory differentiation [87, 88]. The T_CM_ subset is characterized by a delay in production of any prototypic cytokines of the effector lineage after TCR stimulation, a process that initially favors proliferation over differentiation (reviewed in [87]). Activation alone may not effectively differentiate T_CM_ to acquire effector function. Indeed, a previous study observed the T_CM_ subset to be heterogenous in the ability to differentiate and express effector cytokines when activated in non-polarizing conditions *in vitro* [89]. Half of clonal T_CM_ populations activated *in vitro* proliferated in the absence of effector differentiation while other the other half spontaneously differentiated to IFN-γ - or IL- 4-producing cells in the non-polarizing activating conditions through a bona fide stochastic process [89]. Significantly, acetylation profiles of cytokine gene regions showed contrasting patterns in T_CM_ and T_EM_ subsets during *in vitro* non-polarizing activation conditions, with the T_CM_ subset showing hypoacetylation and T_EM_ showing polarized acetylation profiles that correlated with the expression of effector cytokines, supporting the conclusion that T_CM_ and T_EM_ subsets to have differential epigenetic profiles that regulate gene function [89]. Recent studies showed a significant correlation between latency reversal and the upregulation of effector function associated with differentiation. Latency reversing agents that triggered quiescent T_CM_ and T_TM_ to pathways of differentiation correlated with the highest levels of latency reversal [49].

Here, we employed novel enhancements to the previously established QVOA platform to show effector memory differentiation in the presence of γ-chain and DC-derived cytokines significantly induced HIV-1 latency reversal from rCD4+ T cells when coupled with sequential mitogen stimulation and IL-2 exposure. γ-chain cytokines mediate homeostatic proliferation of the T_CM_ and T_EM_ subsets that drive proliferation and maintenance of long-term memory *in vivo*, and exposure to these cytokines can mediate latency reversal in some contexts [73–76]. DC cytokines are known to upregulate the expression of the IL-2/IL15Rβ and γ-chain [90, 91], which supports the CD4+ T cell response to γ-chain cytokines. The level of upregulation of these receptors was comparable to that induced by TCR stimulation, while naïve and memory subsets also were shown to express IL-7R alpha [65]. The DC cytokines also promote greater responsiveness to IL-2, corresponding to the upregulation of IL-2Rα expression we observed in dQVOA. The differentiation effect of γ-chain and DC-derived cytokines is coupled with pro-survival mechanisms such as the upregulation of Bcl-2 that protects T cells from apoptosis [92], thus helping to preserve the viability of cells during differentiation. *In vivo*, pro- and anti-inflammatory signaling pathways mediated by γ-chain and DC-derived cytokines have been shown to effect reservoir size during the establishment of HIV-1 infection [93]. Other cytokines, such as IL-4 and IL-12, which polarize memory CD4+ T cells, may also be employed to induce differentiated subsets that have been shown previously to support high levels of virus production and reverse latency *in vitro* [40, 94]. dQVOA results from two participants, 3068-R and 2026-R, exhibit both a high frequency of the reservoir and cells in the naïve T subset. Recent studies have shown the CD4+ naïve population may contribute significantly to the replication-competent reservoir *in vivo* [95, 96]. Naïve CD4 T cells have been shown to proliferate, upregulate expression of γ-chain cytokine receptors, and express effector cytokines in response to differentiation, albeit to a more limited extent than the T_CM_ subset [65]. These induced pathways also may prime the naïve CD4 T cells to support HIV expression during activation. Alternatively, if the naïve CD4+ T cell population induces robust HIV latency reversal through mitogen activation, the standard and differentiation QVOA conditions both use PHA plus IL-2 stimulation in the presence of allogeneic feeder cells to support the induction of HIV expression. Future studies will examine the memory subset-specific effect of differentiation and polarization on latency reversal and characterize mechanisms of HIV-1 persistence.

The presence of DC derived cytokines enhances the homeostatic proliferation effect of the gamma chain cytokines, and we expect CD4+ T cells to expand in response to differentiation cytokines. However, proliferation alone is not effective at inducing HIV expression. Moso et al. recently demonstrated HIV latency is effectively maintained in proliferating cells [97]. Interestingly, Vandergeeten et al. also showed that the γ-chain cytokine IL-7 does not induce HIV latency reversal in latently infected cells, but can support enhanced HIV expression when the cells are productively infected [74]. Therefore, we would not expect the differentiation step to be a significant driver of latency reversal through proliferation. However, one explanation to the observation of a detectable frequency of HIV post-differentiation (Fig 2B) is that cells with a baseline low level expression of HIV transcripts may be induced into virus production in the presence of IL-7, which in the anti-viral free context of the culture conditions would result in virus propagation and spreading infection to neighboring CD4+ T cells.

Standard QVOA culturing conditions [67] utilize multiple rounds of cellular activation to induce latency reversal. HIV-infected participant derived resting CD4+ T cells initially are introduced into culture with PHA plus IL-2 in the presence of irradiated allogeneic feeder cells from a mixture of two HIV-naïve donors. The following day, a second activation is induced by the addition of activated allogeneic target cells from four unrelated HIV-naïve donors in the presence of high levels of IL-2 (100 U/mL). Alloreactivity is a well-established method of cellular activation, including the memory T cell compartment [98]. Standard QVOA cultures are then subsequently stimulated one more time by the addition of activated allogeneic target cells from a mixture of four additional unrelated HIV-naïve donors on day 8, for a total of 3 rounds of repeated activation of the HIV-infected CD4+ T cell population. dQVOA culture conditions initiate differentiation cytokine culture on day -7, and PHA plus IL-2 activation in the presence of irradiated allogeneic feeder cells from a mixture of two HIV-naïve donors on day 0. No activated allogeneic target cells are added at any point in the culture to induce a third round of activation. The standard QVOA conditions maintain continued and supported activation for almost three weeks in culture and as demonstrated previously fail to effectively induce HIV latency reversal in a significant fraction of the CD4+ population infected with intact provirus [47], and indeed recent attempts to increase the assay sensitivity relies upon long term repeated restimulation to capture the inducible intact proviral reservoir (MS-VOA [48]).

Differentiation is a biological pathway in response to DC signaling aimed at inducing effector cells, including the expansion of the effector cell population, which are part of the required adaptive immune response. T cell differentiation results in the upregulation of the expression of transcription factors such as NF-AT, NF-KB and AP-1, all factors previously identified to be associated with HIV expression [51, 99, 100]. These transcription factor complexes are involved in chromatin remodeling and expression of lineage specific cytokines [101]. We compare activation alone (standard QVOA) to the combination of differentiation followed by activation (dQVOA) to understand how differentiation signals influence HIV latency reversal. We find that polarizing resting CD4+ T cells through effector differentiation followed by mitogen activation results in a higher frequency of HIV latency reversal, as measured by a significantly increased IUPM. The combination of proliferation, activation and acquisition of effector function that directly result from differentiation may all contribute specifically to the ability of resting CD4+ T cell populations to support the induction of HIV expression that cannot be as effectively obtained upon mitogen stimulation alone. Interestingly, although we observed an increased IUPM frequency in all participants with a detectable replication-competent reservoir (Fig 1F), the fold increase was variable. This observation may reflect the dynamics of the reservoir within each individual, which includes factors such as the prevalence of expanded clones and immunological signatures that stabilize the reservoir. Future studies will examine the influence of inflammatory signatures that drive HIV persistence in different resting CD4+ T cell subsets, and how these signatures may influence APC differentiation responses. Together, these data demonstrate the impact of differentiation over activation alone for efficient latency reversal, specifically in the context of the HIV-1 latent reservoir, and provide mechanistic insight into the stochastic reactivation observed in QVOA. Further characterizing these mechanisms will provide the basis for understanding the dynamics that influence latency reversal in highly-quiescent, memory CD4+ T cell compartments *in vivo*, as well as identifying potential mechanisms for viral rebound during treatment interruption.

Quantifying the number of cells harboring latent, replication-competent HIV-1 provirus is critical to evaluating cure strategies, but the low frequency and accumulation of inactivating mutations makes precise quantification extremely challenging through molecular-based approaches. Our data demonstrates that differentiation of rCD4+ cells prior to QVOA is an effective tool for promoting latency reversal *in vitro*, which enhances the performance of existing protocols to quantify the frequency of replication-competent HIV-1 in rCD4+ T cells from virally-suppressed individuals. Results from recent assays quantifying the intact proviral reservoir have suggested the frequency of non-mutated genomes is much higher than what has been estimated by viral outgrowth [102]. However, numerous host cell as well as virologic factors may contribute to viral replication fitness *in vivo*, and understanding the mechanisms that direct HIV expression from latently infected cells remains a critical component both in reservoir quantification and studying HIV persistence. For the first time, *ex vivo* effector memory differentiation has moved reservoir measurements closer toward what may be the bona fide replication-competent reservoir frequency, thus beginning to bridge the gap between outgrowth and molecular-based quantification. Taken together, these data support accumulating evidence that effector memory differentiation is a key pathway to HIV-1 latency reversal that may be exploited for assay development, mechanistic understanding, and therapeutic interventions.

## Materials and methods

### Study participants

PBMC samples were obtained from 12 HIV-1 infected, virally suppressed adult male participants of two established study cohorts. Two participants were in the NIH cohort Analysis of HIV-1 Replication During Antiretroviral Therapy (AVBIO2, protocol 08-I-0221) and ten participants were in the HIV Reservoir Assay Validation and Evaluation Network (RAVEN) cohort. All participants initiated ART during chronic infection and exhibited viral suppression in plasma HIV-1 VL (<40 copies/mL) for greater than 10 years. Plasma HIV-1 VL and T cell measurements were performed at each study visit.

### Ethics statement

The NIH study was approved by the NIAID institutional review board (IRB FWA#00005897) and the RAVEN cohort was approved by the UCSF Committee on Human Research (IRB #10–03244). RAVEN participants are enrolled and followed as part of the UCSF OPTIONS and SCOPE programs with specific consent for apheresis collections and testing for this study. All participants were adults (>18 years of age) and provided written, informed consent.

### QVOA

QVOA was performed as previously described with minor revision[67]. Briefly, cryopreserved PBMC from HIV-1-infected participants were thawed and cultured overnight in complete media (RPMI-1640 with Glutamax(Gibco, 61870–036) containing 10% heat-inactivated fetal bovine serum (Peak Serum, PS-FB1), 1% penicillin and 1% streptomycin (Gibco, 15140–122)). Cells were enriched for rCD4+ T cells through negative, magnetic bead separation (StemCell, 17962 and 19250) and assessed for purity and memory T cell subsets by flow cytometric analysis. rCD4+ T cells were plated at 1x10^6^ cells per well (A dilution), 2x10^5^ cells per well (B dilution), and 4x10^4^ cells per well (C dilution) in complete media with the addition of 100 IU/mL recombinant human IL-2 (R&D Systems, 202-IL), 1–1.2% T cell growth factor (TCGF) prepared as described previously[67], and 0.5 μg/mL PHA (ThermoFisher Scientific, Remel, R30852801). γ-irradiated allogeneic PBMC were added at a 10:1 ratio. Following overnight incubations, PHA was diluted in the cultures through media replacement and CD8-depleted (ThermoFisher Scientific, 11147D) lymphoblasts were added as targets for viral expansion. Media and cells were replenished and replaced according to previous publication and maintained for up to 20 days. All supernatants removed for nutrient supplementation were tested for the presence of p24 antigen by ELISA (PerkinElmer, NEK050B001KT) according to the kit protocol. Wells were considered positive for HIV-1 outgrowth if the p24 concentration was above 3.25 pg/mL. IUPMStats v1.0 was used to calculate the frequency of latently infected, induced rCD4+ T cells[66].

### Differentiation QVOA (dQVOA)

A detailed protocol can be found on protocols.io [103]. As described above for QVOA, cryopreserved PBMC were thawed, rested overnight, and magnetically enriched for rCD4+ T lymphocytes following manufacturer protocol (StemCell, 17962 and 19250). For all RAVEN participants, QVOA and dQVOA were initiated in parallel from the same original pool of rCD4+ T cells. After magnetic enrichment and before differentiation, rCD4+ T cells were diluted and plated at 5x10^5^ cells per well (A dilution), 2x10^5^ cells per well (B dilution), 4x10^4^ cells per well (C dilution), and 8x10^3^ cells per well (D dilution) in complete media containing 25 ng/mL each of TNF-α, IL-6, IL-7, IL-10, and IL-15 (R&D Systems, 210-TA, 206-IL, 207-IL, 217-IL, 247-ILB respectively). Media and cytokines were replenished every 3–4 days by replacing half of the media without disturbing the cellular layer. After 6–7 days of differentiation and without removing exogenous cytokines, individual wells were stimulated with 0.5 μg/mL PHA, 100 IU/mL IL-2, and γ-irradiated allogeneic PBMC at a 10:1 ratio. Following overnight incubation, PHA concentrations were reduced through replacement of stimulation media with complete media containing 100 IU/mL IL-2 in cultures. Cultures were replenished every 3–4 days by replacing half of the complete media containing 100 IU/mL IL-2 without disturbing the cellular layer. As with QVOA, all supernatants from media replenishment and end of assay time points were tested for the presence of p24 antigen by ELISA (PerkinElmer, NEK050B001KT) according to the kit protocol and IUPMs were calculated with IUPMStats v1.0[66].

### Flow cytometry

During rCD4+ T cell enrichment and differentiation, cells were assessed for memory phenotype through flow cytometric analysis and as previously published [65]. The antibodies were obtained from Biolegend unless otherwise noted: CD3 (HIT3a, Pacific Blue, 300330), CD4 (RPA-T4, Brilliant Violet 510, 300546), CD8 (SK1, PerCP-Cy5.5, 344710), CD16 (eBioscience, eBioCB16, FITC, 11-0168-42), CD24 (eBioscience, eBioSN3 A5-2H10, FITC, 11-0247-42), CD25 (BD Biosciences, M-A251, APC, 555434), CD45RA (HI100, APC-Cy7, 304128), CD27 (BD Biosciences, L128, PE, 340425), CCR7 (BD Biosciences, 3D12, PE-Cy7, 557648). A lineage (Lin) cocktail included antibodies against CD16 and CD24. Cell viability was assessed via the use of a Live/Dead viability kit (Invitrogen, FITC, L34970). Data was collected with a Stratedigm S1000Exi flow cytometer and analyzed on FlowJo v10.0 software (BD Biosciences). RCD4+ T cells were identified as CD3^+^CD8^–^Lin^–^CD25^–^. Naïve CD4+ T cells were identified as CD3^+^CD8^–^Lin^–^CD45RA^+^CCR7^+^CD27^+^. T_CM_ were identified as CD3^+^CD8^–^Lin^–^CD45RA^–^CCR7^+^CD27^+^. T_TM_ were identified as CD3^+^CD8^–^Lin^–^CD45RA^–^CCR7^–^CD27^+^. T_EM_ were identified as CD3^+^CD8^–^Lin^–^CD45RA^–^CCR7^–^CD27^–^. T_EMRA_ were identified as CD3^+^CD8^–^Lin^–^CD45RA^+^CCR7^–^CD27^–^.

### Single genome sequencing

Single-genome sequencing (SGS) of a portion of p6-PR-RT was performed as previously described [9]. Sequences were aligned using ClustalW. Neighbor-joining phylogenetic analyses were performed using MEGA7.

### Statistical analyses

Due to differing antibody panels and flow cytometric gating strategies, NIH2-20 and NIH2-37 were excluded from correlation analyses involving T cell subsets. When an assay failed to yield a p24 positive well, the assay-specific maximum likelihood value for zero positive wells was used in statistical analyses. Wilcoxon matched-pairs signed rank and t tests were performed with GraphPad Prism version 8.0.0. Correlation analyses were carried out using Python v.3.3.1 with the Scipy v.1.1.0 library using the Pearson correlation coefficient (r value). Statistical significance of linear regression was determined using the two-sided hypothesis Wald Test with t-distribution built in to the Scipy *linregress* module. Sequence Overrepresentation (SOR) Index was determined using the tool available at (https://michaelbale.shinyapps.io/prob_identical/).

## Supporting information

S1 Fig *Ex vivo* effector memory differentiation upregulates CD25 expression on all memory T cell subsets in dQVOA. a, Column plot showing CD25 expression on either naïve or memory T cell subsets. Wilcoxon matched-pairs signed rank tests were used and ** denotes p < 0.01. b, Column plots showing percentages of memory T cells staining positive for IFN-g+, IL-4+ and IFN-g+/IL-4+ and the fold-increases of the percentage memory T cells expressing CCR5+ after differentiating for 7 days. Samples were evaluated from three separate uninfected (UI) and HIV-1-infected, virally suppressed individuals (HIV+) according to previous publication [65]. Each independent sample is shown, with the grand mean and standard deviation shown. Wilcoxon matched-pairs signed rank tests were used and NS denotes insignificant differences between groups.
(TIF)

S2 Fig dQVOA generates higher IUPM values with or without target lymphoblast addition. rCD4+ T cells from 3 independent virally suppressed participants were evaluated using standard QVOA (red circles), dQVOA + lymphoblast targets (blue squares), and dQVOA (no lymphoblast targets added; green triangles) to generate IUPM values. Error bars represent 95% confidence intervals. BD = below detection.
(TIF)

S1 Table Frequency of HIV-GAG+ wells in each dilution. Number of p24+ positive wells over the total number of wells plated per assay is shown for dQVOA (grey banded rows) versus QVOA (white banded rows). ^1^Dilution A in QVOA is 1x10^6^ rCD4+ T cells per well and dQVOA is 5x10^5^ rCD4+ T cells per well. Dilutions B through E are consistent between the two assays. NA, not applicable.
(PDF)
