# Supplementary figures and images for: Effector memory differentiation increases detection of replication-competent HIV-l in resting CD4+ T cells from virally suppressed individuals
Source: PLoS Pathog. 2019 Oct 14;15(10):e1008074. doi: 10.1371/journal.ppat.1008074 (PMC6812841; doi:10.1371/journal.ppat.1008074)

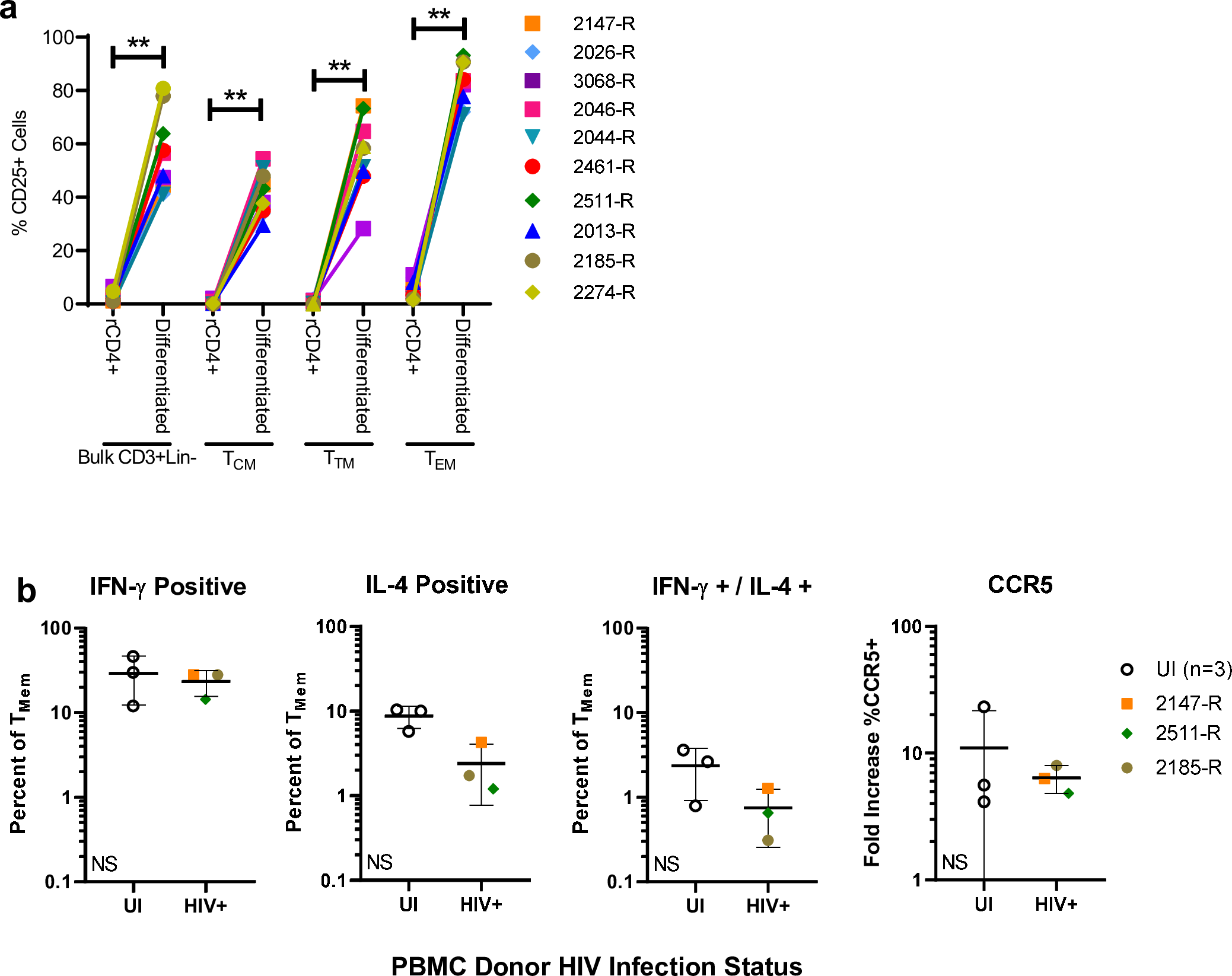

Supplement: S1 Fig — a, Column plot showing CD25 expression on either naïve or memory T cell subsets. Wilcoxon matched-pairs signed rank tests were used and ** denotes p < 0.01. b, Column plots showing percentages of memory T cells staining positive for IFN-g+, IL-4+ and IFN-g+/IL-4+ and the fold-increases of the percentage memory T cells expressing CCR5+ after differentiating for 7 days. Samples were evaluated from three separate uninfected (UI) and HIV-1-infected, virally suppressed individuals (HIV+) according to previous publication [65]. Each independent sample is shown, with the grand mean and standard deviation shown. Wilcoxon matched-pairs signed rank tests were used and NS denotes insignificant differences between groups. (TIF) [file ppat.1008074.s001.tif]

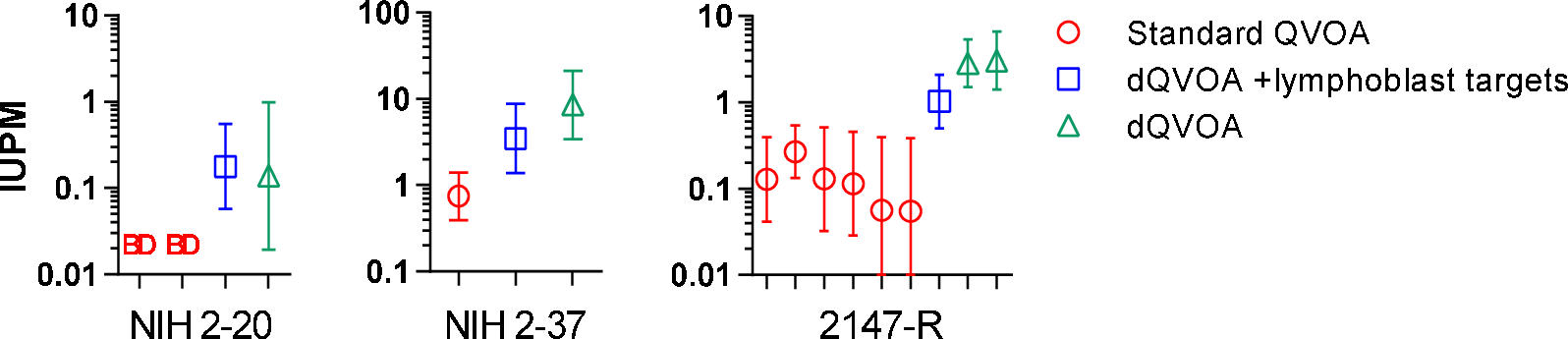

Supplement: S2 Fig — rCD4+ T cells from 3 independent virally suppressed participants were evaluated using standard QVOA (red circles), dQVOA + lymphoblast targets (blue squares), and dQVOA (no lymphoblast targets added; green triangles) to generate IUPM values. Error bars represent 95% confidence intervals. BD = below detection. (TIF) [file ppat.1008074.s002.tif]
