# Supplementary material for: Effector memory differentiation increases detection of replication-competent HIV-l in resting CD4+ T cells from virally suppressed individuals
Source: PLoS Pathog. 2019 Oct 14;15(10):e1008074. doi: 10.1371/journal.ppat.1008074 (PMC6812841; doi:10.1371/journal.ppat.1008074)
Supplement: S1 Table — Number of p24+ positive wells over the total number of wells plated per assay is shown for dQVOA (grey banded rows) versus QVOA (white banded rows). 1Dilution A in QVOA is 1x10^6^ rCD4+ T cells per well and dQVOA is 5x10^5^ rCD4+ T cells per well. Dilutions B through E are consistent between the two assays. NA, not applicable. (PDF) [file ppat.1008074.s003.pdf]

S1 Table. Frequency of HIV-GAG+ wells in each dilution dQVOA vs QVOA

Participant ID	dilution A[1] (+ wells/ total wells)	dilution B (+ wells/ total wells)	dilution C (+ wells/ total wells)	dilution D (+ wells/ total wells)	dilution E (+ wells/ total wells)	Infectious Units Per Million (IUPM)
NIH 2-20	1+/12	0+/7	0+/2	NA	NA	0.138
	0+/24	0+/2	0+/2	0+/2	0+/2	BD (0.028)
NIH 2-37	8+/8	2+/3	1+/2	NA	NA	8.595
	9+/18	0+/2	0+/2	0+/2	1+/2	0.744
2147-R	11+/12	2+/5	0+/2	0+/2	NA	3.986
	1+/12	0+/2	0+/2	NA	NA	0.084
2026-R	2+/3	4+/9	0+/2	0+/2	NA	2.543
	0+/12	0+/2	0+/2	NA	NA	BD (0.056)
3068-R	5+/8	2+/12	0+/2	0+/2	NA	1.432
	1+/12	0+/2	0+/2	NA	NA	0.084
2046-R	9+/12	2+/5	0+/2	0+/2	NA	2.654
	2+/12	0+/2	0+/2	NA	NA	0.175
2044-R	4+/12	2+/5	0+/2	0+/2	NA	1.034
	5+/12	1+/2	0+/2	NA	NA	0.624
2461-R	0+/12	0+/5	0+/2	0+/2	NA	BD (0.098)
	0+/12	0+/2	0+/2	NA	NA	BD (0.056)
2511-R	9+/12	3+/5	0+/2	0+/2	NA	3.023
	8+/12	1+/2	1+/2	NA	NA	1.335
2013-R	0+/12	0+/5	0+/2	0+/2	NA	BD (0.098)
	0+/12	0+/2	0+/2	NA	NA	BD (0.056)
2185-R	10+/12	3+/5	0+/2	0+/2	NA	3.680
	3+/12	0+/2	0+/2	NA	NA	0.275
2274-R	9+/9	5+/5	0+/2	1+/2	NA	17.218
	11+/12	1+/2	0+/2	NA	NA	2.510
